# Supplementary material for: Synthesis and Characterization of Vanillin-Based π-Conjugated Polyazomethines and Their Oligomer Model Compounds
Source: Molecules. 2022 Jun 28;27(13):4138. doi: 10.3390/molecules27134138 (PMC9268122; doi:10.3390/molecules27134138)
Supplement: Supplementary file 1 [file molecules-27-04138-s001.zip › molecules-1715416-supplementary.pdf]

Supplementary Materials

Synthesis and Characterization of Vanillin-Based π-Conjugated Polyazomethines and Their Oligomer Model Compounds

Lauriane Giraud [1], Stéphane Grelier [1], Etienne Grau [1], Laurent Garel [2], Georges Hadziioannou [1], Brice Kauffmann [3], Éric Cloutet [1], Henri Cramail [1,*] and Cyril Brochon [1,*]

[1] University of Bordeaux, CNRS, Bordeaux INP, LCPO, UMR5629, 33600 Pessac, France

[2] Solvay—GBU Aroma Performance, Recherche & Innovation—Centre de Lyon (RICL), 85 Rue des frères Perret, 69192 Saint Fons, France

[3] Institut Européen de Chimie Biologie (UAR3033/US001), University of Bordeaux, CNRS, INSERM, 2 Rue Escarpit, 33600 Pessac, France

***** Correspondence: henri.cramail@enscbp.fr (H.C.); cyril.brochon@enscbp.fr (C.B.)

Table of content

Figure S1: ^{1}H-NMR spectrum of purified **DV** (400.20 MHz, in DMSO-d6) 4

Figure S2: ^{13}C-NMR spectrum of purified **DV** (100.70 MHz, in DMSO-d6) 4

Figure S3: ^{1}H-NMR spectrum of **DVM** (400.20 MHz, in $CDCl_3$) 5

Figure S4: ^{13}C-NMR spectrum of **DVM** (100.70 MHz, in $CDCl_3$) 5

Figure S5: ^{1}H-NMR spectrum of **DVEH** (400.20 MHz, in $CDCl_3$) 5

Figure S6: ^{13}C-NMR spectrum of **DVEH** (100.70 MHz, in $CDCl_3$) 6

Figure S7: ^{1}H-NMR spectrum of **P1** (400.20 MHz, in $CDCl_3$, 128 scans) 6

Figure S8: a. SEC trace of **P1** in THF, R. I. detection. **b.** TGA curve of P1 at 10 °C/min under N_2 until 650 °C, then under air 7

Figure S9: ^{1}H-NMR spectrum of **P2** (400.20 MHz, in $CDCl_3$, 128 scans) 7

Figure S10: a. SEC trace of **P2** in THF, R. I. detection. **b:** TGA curve of **P2** at 10 °C/min under N_2 8

Figure S11: ^{1}H-NMR spectrum of **P3** (400.20 MHz, in $CDCl_3$, 128 scans) 8

Figure S12: **a.** SEC trace of **P3** in THF, R. I. detection. **b:** TGA curve of **P3** at 10 °C/min under N_2 9

Figure S13: ^{1}H-NMR spectrum of **P4** (400.20 MHz, in $CDCl_3$, 128 scans) 9

Figure S14: a. SEC trace of **P4** in THF, R. I. detection. **b:** TGA curve of **P4** at 10 °C/min under N_2 10

Figure S15: TGA curve of **P5** at 10°C/min under N_2 10

Figure S16: ^{1}H-NMR spectrum of **P6** (400.20 MHz, in $CDCl_3$, 128 scans) 11

Figure S17: a. SEC trace of **P6** in THF, R. I. detection. **b:** TGA curve of **P6** at 10 °C/min under N_2 11

Figure S18: ^{1}H-NMR spectrum of **M1a** (400.20 MHz, in CD_2Cl_2) 12

Figure S19: ^{13}C-NMR spectrum of **M1a** (100.70 MHz, in CD_2Cl_2) 12

Figure S20: ^{1}H-NMR spectrum of **M1b** (400.20 MHz, in DMSO-d6) 12

Figure S21: ^{13}C-NMR spectrum of **M1b** (100.70 MHz, in DMSO-d6) 13

Figure S22: ^{1}H-NMR spectrum of **M2** (400.20 MHz, in CD_2Cl_2) 13

Figure S23: ^{13}C-NMR spectrum of **M2** (100.70 MHz, in CD_2Cl_2) 13

Figure S24: ^{1}H-NMR spectrum of **M3a** (400.20 MHz, in CD_2Cl_2) 14

Figure S25: ^{13}C-NMR spectrum of **M3a** (100.70 MHz, in CD_2Cl_2) 14

Figure S26: ^{1}H-NMR spectrum of **M3b** (400.20 MHz, in DMSO-d6) 14

Figure S27: ^{13}C-NMR spectrum of **M3b** (100.70 MHz, in DMSO-d6) 15

Figure S28: ^{1}H-NMR spectrum of **M4** (400.20 MHz, in CD_2Cl_2) 15

Figure S29: ^{13}C-NMR spectrum of **M4** (100.70 MHz, in CD_2Cl_2) 15

Figure S30: Absorbance spectra of **P1** in methylene chloride (10^{-2} g/L) with different amount of silica and emission spectra of **P1** (10^{-3} g/L, excitation at 360 nm) with and without silica. All solutions were filtered before analysis....16

Figure S31: SEC trace of a **P4** polymer synthesized by only performing a “recovery step” on a stoichiometric mixture of monomers (R.I. detection, in THF) 16
Figure S32: Absorbance spectra of **DVEH** and **DVM** in methylene chloride, 10^{-2} g/L 17
Figure S33: Emission spectra of polyazomethines in methylene chloride and their structure (10^{-2} g/L, which corresponds to an absorbance of 1, soluble fraction for **P5**, integration time: 0.5s)....................................17
Table S1: Absorbance characterizations of model compounds and corresponding polymers.................................19
Table S2 Crystal data and structure refinement for M3b. **Deposition Number 2113008**.....................................19
Table S3 Crystal data and structure refinement for M1a. **Deposition Number 2113009**.....................................20
Table S4 Crystal data and structure refinement for M3a. **Deposition Number 2113010**.....................................21
Table S5 Crystal data and structure refinement for M4. **Deposition Number 2113011**.......................................22
Table S6 Crystal data and structure refinement for M1b. **Deposition Number 2113012**.....................................23
Table S7 Crystal data and structure refinement for M2. **Deposition Number 2113013**.......................................24

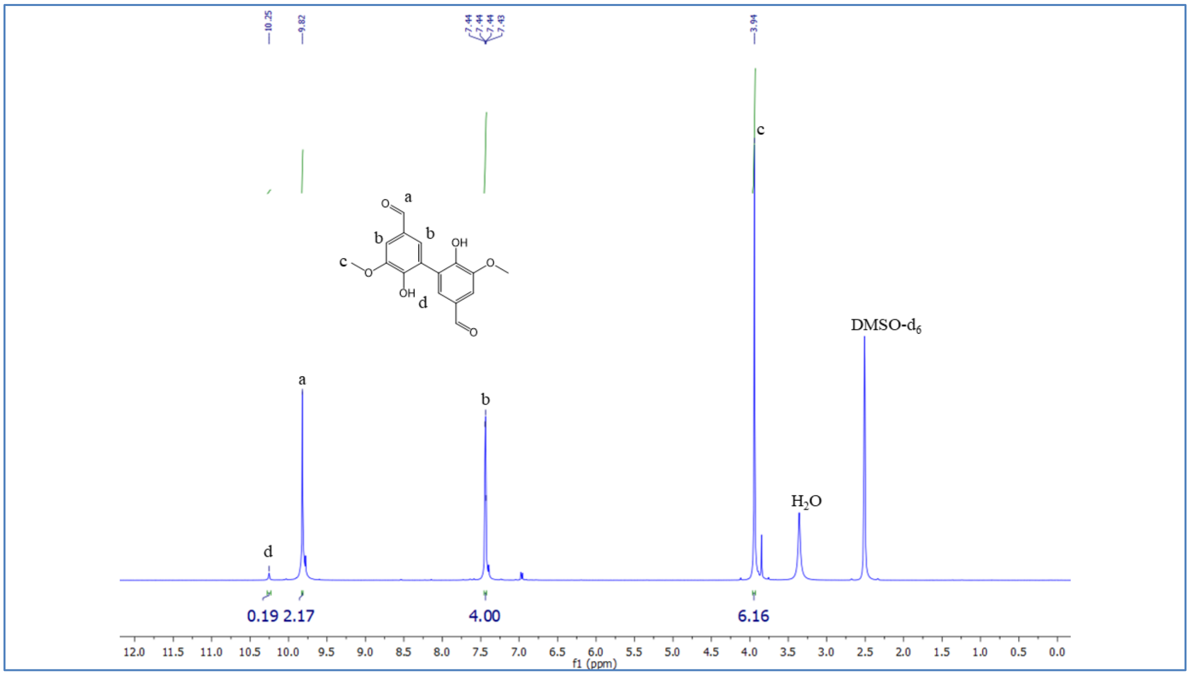

Figure S1: ^{1}H NMR spectrum of purified **DV** (400.20 MHz, in DMSO-d_6).

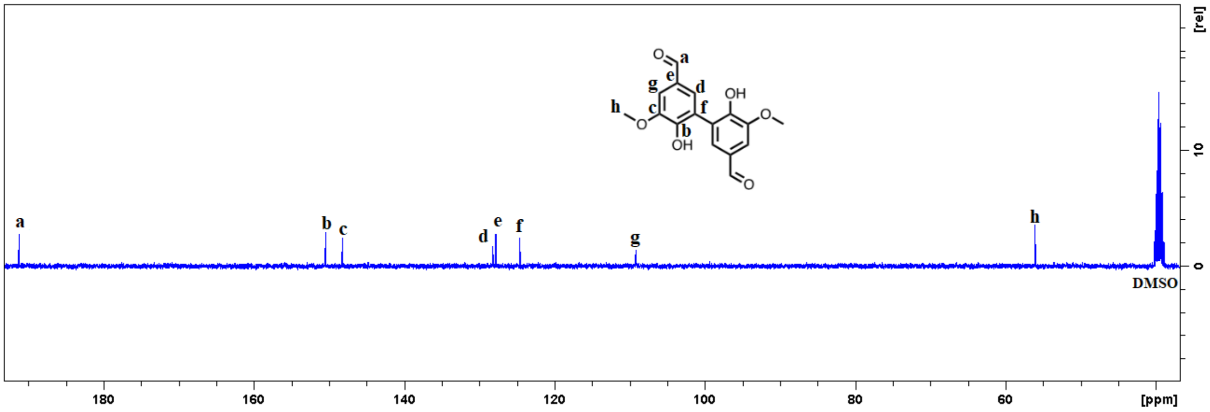

Figure S2: ^{13}C NMR spectrum of purified **DV** (100.70 MHz, in DMSO-d_6).

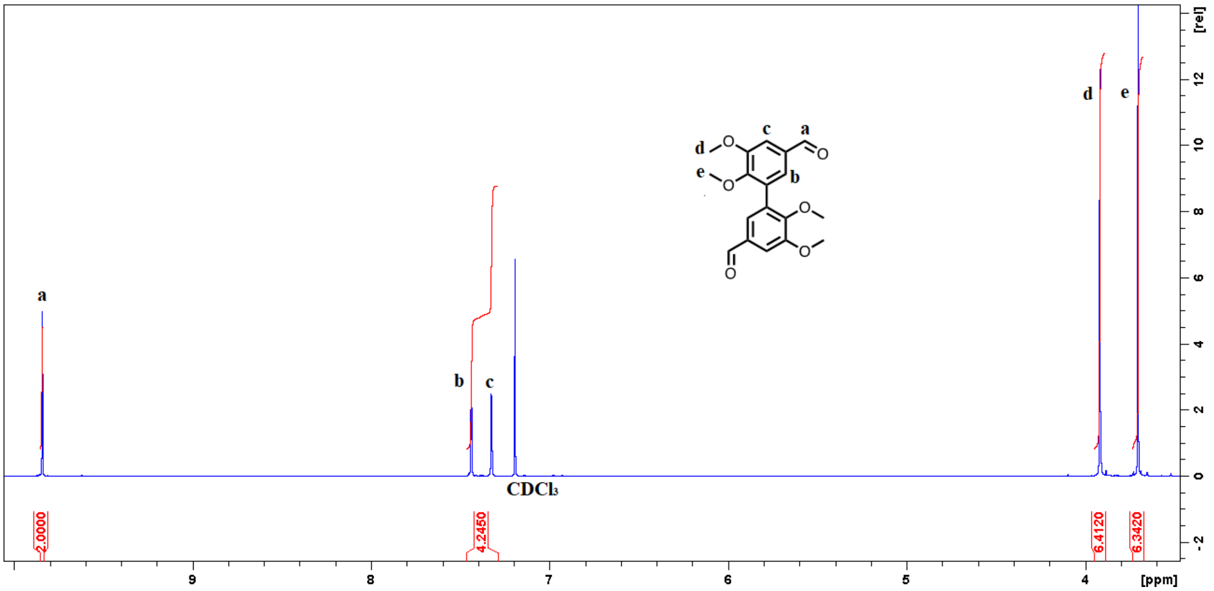

Figure S3: ^{1}H NMR spectrum of **DVM** (400.20 MHz, in $CDCl_3$).

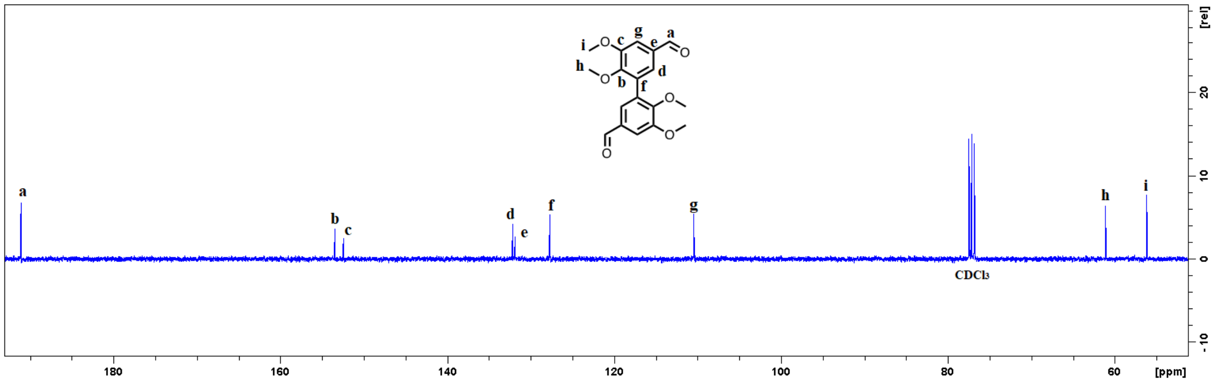

Figure S4: ^{13}C NMR spectrum of **DVM** (100.70 MHz, in $CDCl_3$).

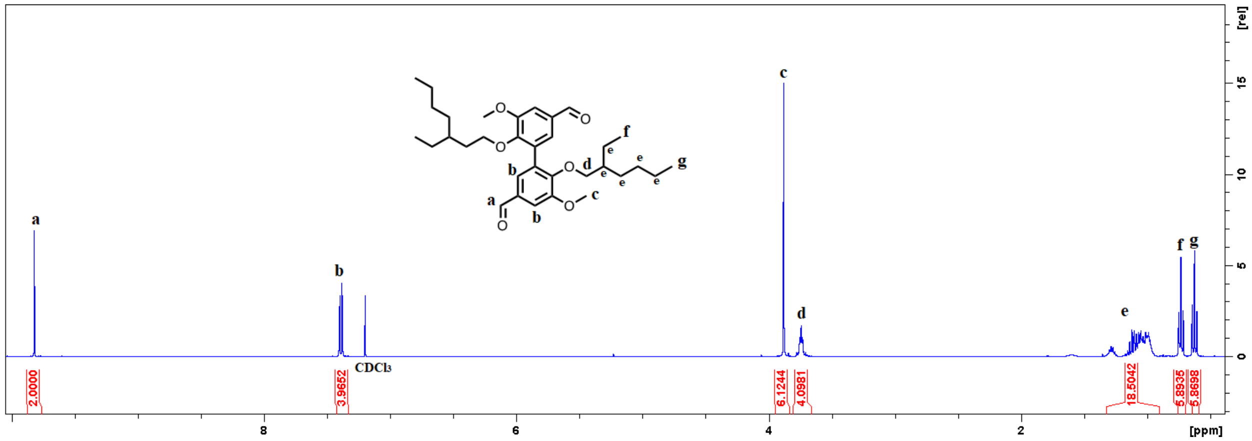

Figure S5: ^{1}H NMR spectrum of **DVEH** (400.20 MHz, in $CDCl_3$).

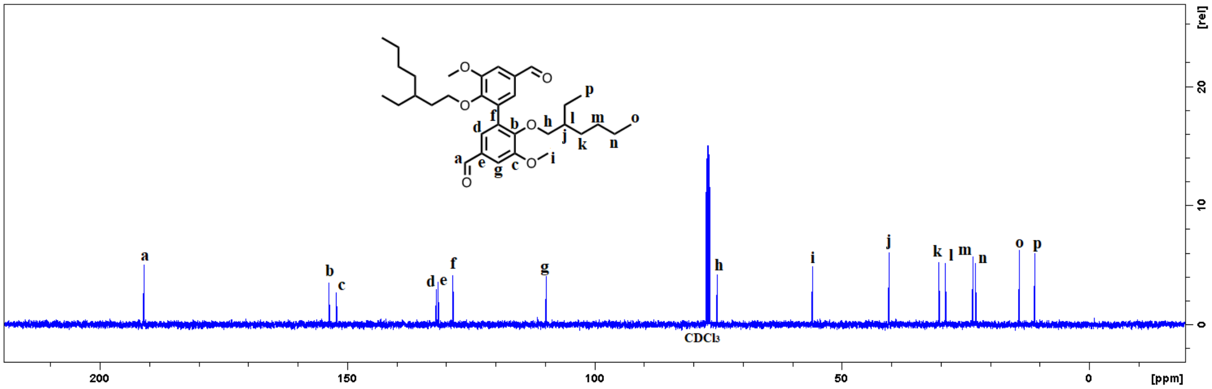

Figure S6: ^{13}C NMR spectrum of **DVEH** (100.70 MHz, in $CDCl_3$).

Characterization of P1

Poly(6,6′-2-ethylhexoxy-5,5′-dimethoxy-[1,1′-biphenyl] – 3,3‘-methylidene- (9,9 dioctyl *n,n*-fluorenylamine

^{1}H NMR (400.2 Hz, $CDCl_3$): 9.89 (s, 0.043H), 8.48 (s, 2H), 7.69 (m, 3.9H), 7.41 (m, 1.78H), 7.21 (s, 4.67H), 4.00 (s, 5.67H), 3.80–3.79 (d, *J* = 4 Hz, 3.9H), 2.10–1.69 (m, 11H), 1.49–0.93 (m, 44H), 0.85–0.72 (m, 19H).

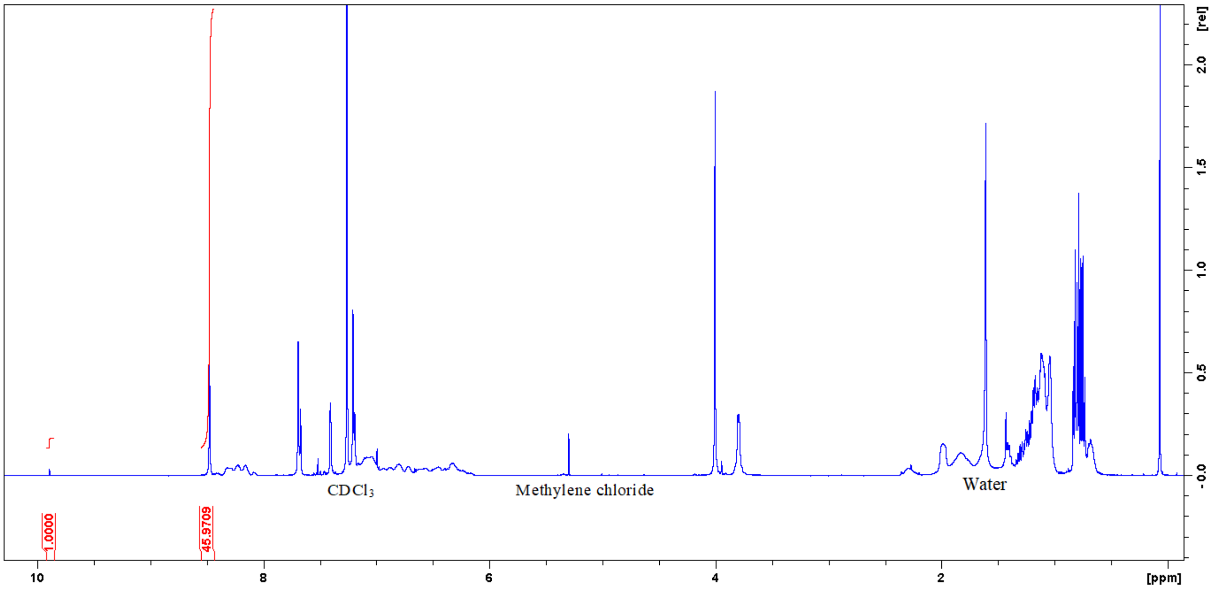

Figure S7: ^{1}H NMR spectrum of **P1** (400.20 MHz, in $CDCl_3$, 128 scans)

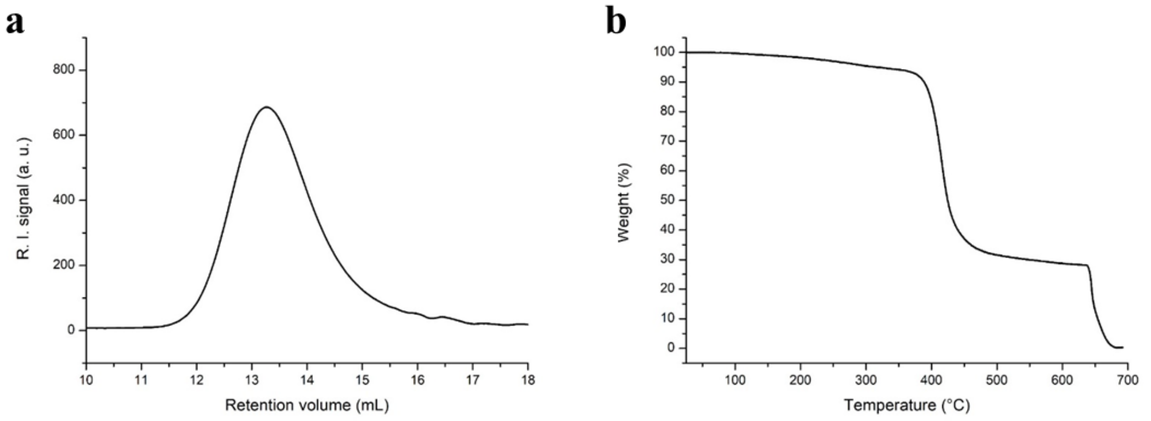

Figure S8: a) SEC trace of **P1** in THF, R. I. detection. **b)** TGA curve of P1 at 10 °C/min under N_2 until 650 °C, then under air.

Characterization of P2

Poly(6,6'-methoxy-5,5'-dimethoxy-[1,1'-biphenyl]-3,3'-methylidene-(9,9-dioctyl-n,n-fluorenylamine))

^{1}H-NMR (400.2 Hz, $CDCl_3$): 9.92 (s, 0.064H), 8.50 (s, 2H), 7.72–7.67 (m, 3.8H), 7.36 (m, 1.78H), 7.22 (s, 3.4H), 4.04 (s, 5.8H), 3.77 (s, 6.2H), 2.05-1.9 (m, 3.8H), 1.27–0.98 (m, 23.2H), (d, *J* = 4 Hz, 3.9H), 2.10–1.69 (m, 11H), 1.49–0.93 (m, 44H), 0.85–0.57 (m, 11.3H).

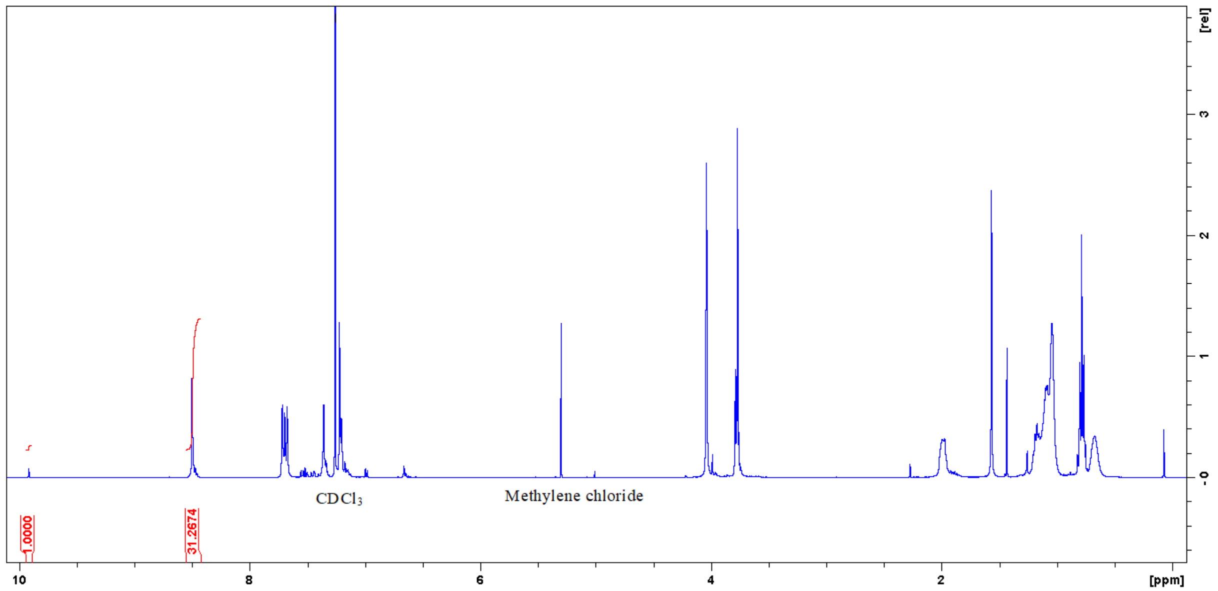

Figure S9: ^{1}H NMR spectrum of **P2** (400.20 MHz, in $CDCl_3$, 128 scans)

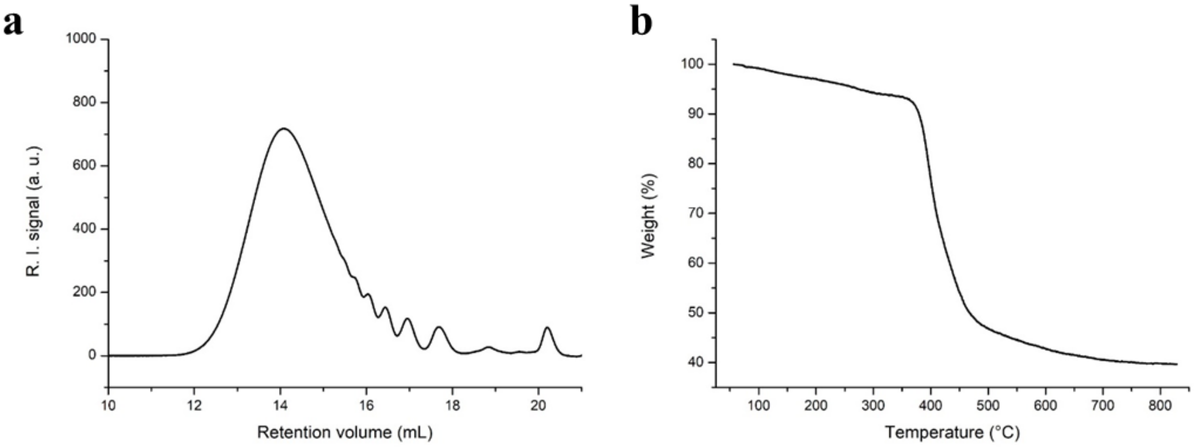

Figure S10: a) SEC trace of **P2** in THF, R. I. detection. **b)** TGA curve of **P2** at 10 °C/min under N_2.

Characterization of P3

Poly(6,6′-2-ethylhexoxy-5,5′-dimethoxy-[1,1′-biphenyl]-3,3‘-methylidene-(1,4-benzene diamine)

^{1}H-NMR (400.2 Hz, $CDCl_3$): 9.89 (s, 0.024H), 8.42 (s, 2H), 7.67 (m, 1.9H), 7.36 (m, 1.6H), 3.99 (s, 6.4H), 3.77 (s, 4H), 1.58 (s, 1.5H), 1.45–1.01 (m, 20H), 0.81 (t, J = 8 Hz, 6.2H), 0.73 (t, J = 8 Hz, 6.2H). (NB: the four protons of the phenylene moiety are superposed with the solvent peak).

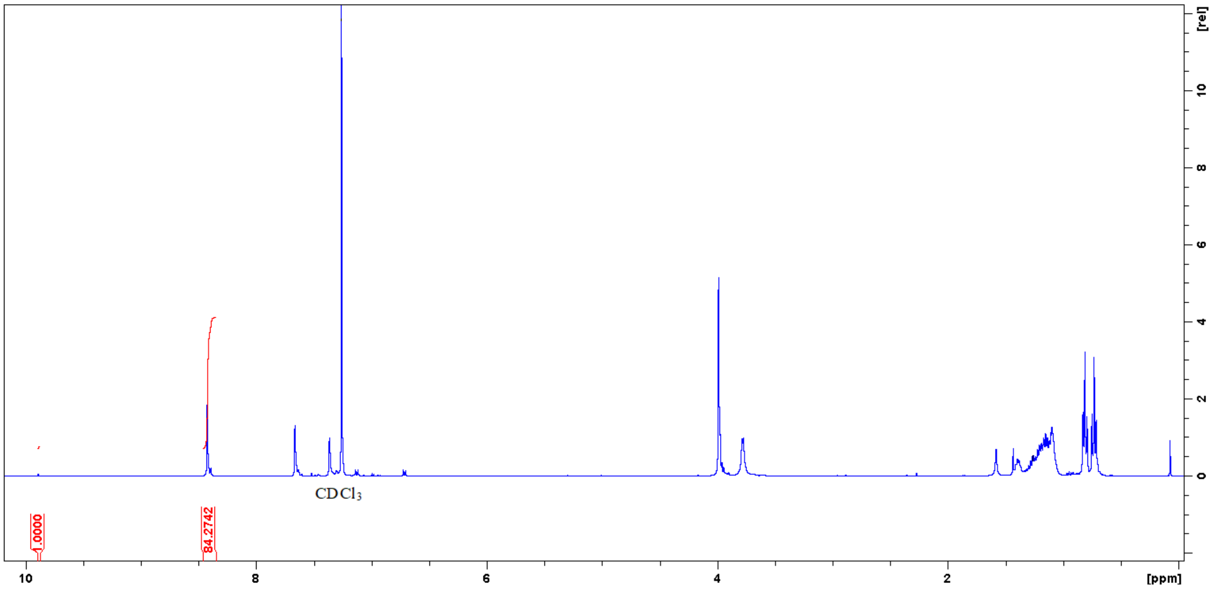

Figure S11: ^{1}H NMR spectrum of **P3** (400.20 MHz, in $CDCl_3$, 128 scans).

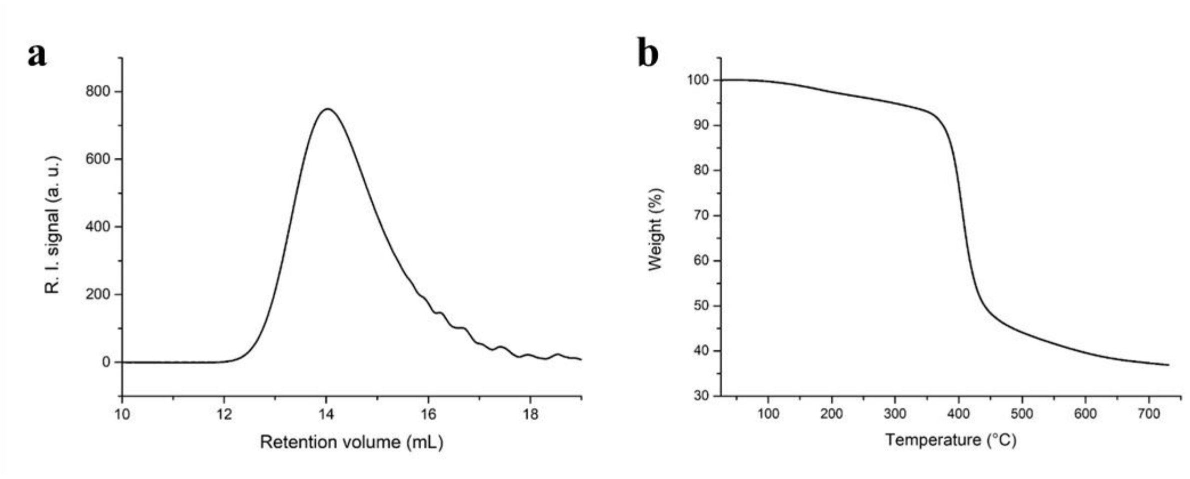

Figure S12: **a)** SEC trace of **P3** in THF, R. I. detection. **b)** TGA curve of **P3** at 10 °C/min under N_2.

Characterization of P4

Poly(6,6'-methoxy-5,5'-dimethoxy-[1,1'-biphenyl]-3,3'-methylidene-(1,4-benzene diamine)

^{1}H-NMR (400.2 Hz, $CDCl_3$): 9.91 (s, 0.092H), 8.44 (s, 2H), 7.68 (s, 1.9H), 7.31 (m, 1.6H), 4.02 (s, 6.2H), 3.75 (s, 6.5H).

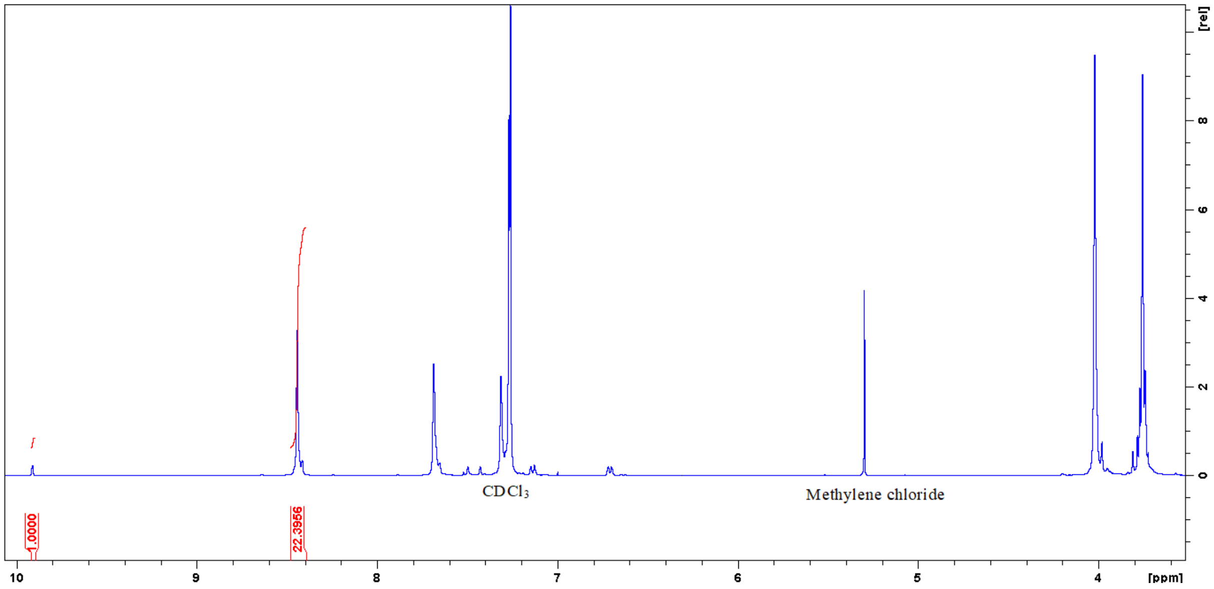

Figure S13: ^{1}H NMR spectrum of **P4** (400.20 MHz, in $CDCl_3$, 128 scans).

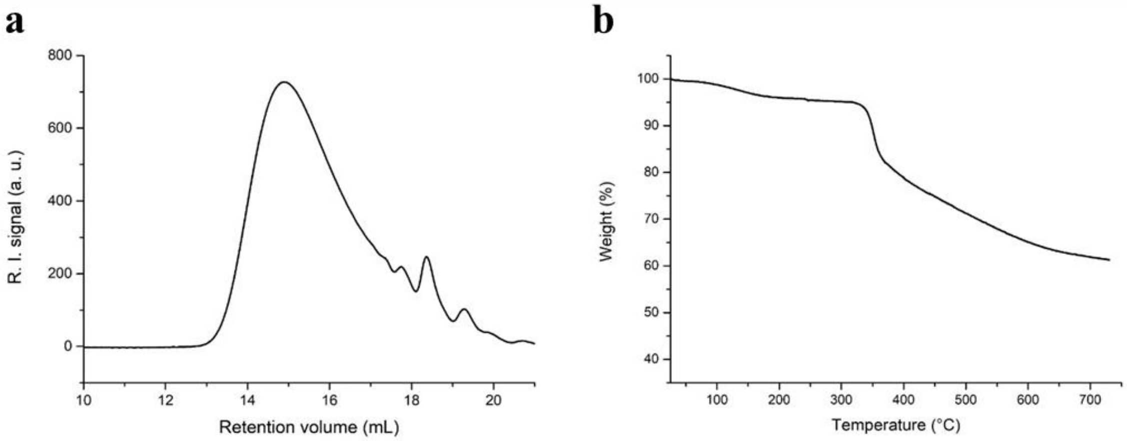

Figure S14: a) SEC trace of **P4** in THF, R. I. detection. **b)** TGA curve of **P4** at 10 °C/min under N_2.

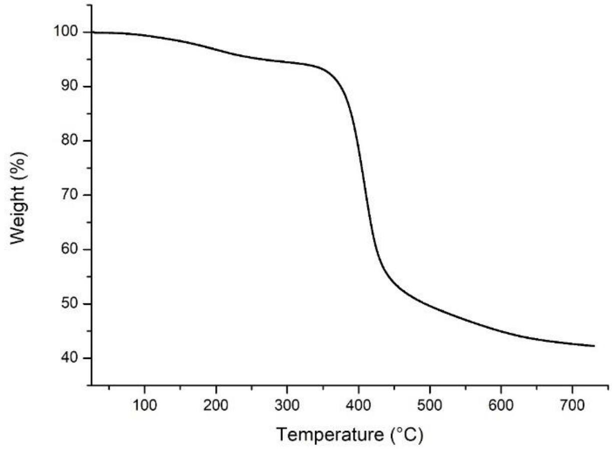

Figure S15: TGA curve of **P5** at 10 °C/min under N_2.

Characterization of P6

Poly (6,6′-methoxy-5,5′-dimethoxy-[1,1′-biphenyl]-3,3‘-methylidene-(1,3benzene diamine)

^{1}H-NMR (400.2 Hz, $CDCl_3$): 9.91 (s, 1H), 8.44 (s, 2.45H), 7.68 (s, 2.45H), 7.49 (m, 1.1H), 7.43–7.36 (m, 2.42H), 7.10–7.02 (m, 4.18H), 4.04–3.96 (m, 12H), 3.82–3.71 (m, 13.8H).

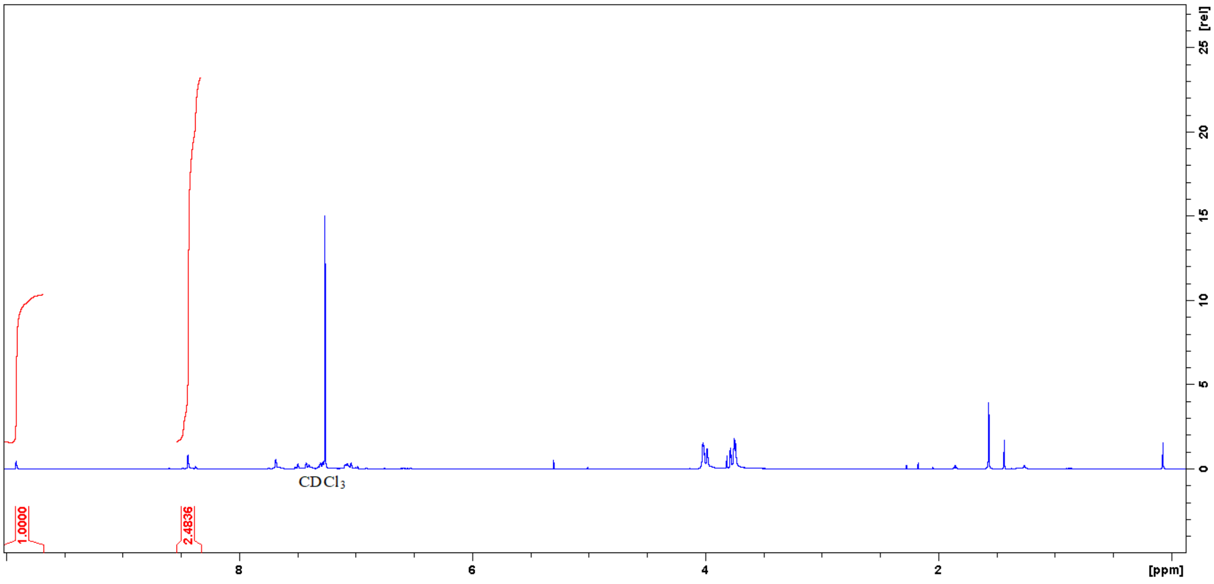

Figure S16: ^{1}H NMR spectrum of **P6** (400.20 MHz, in $CDCl_3$, 128 scans).

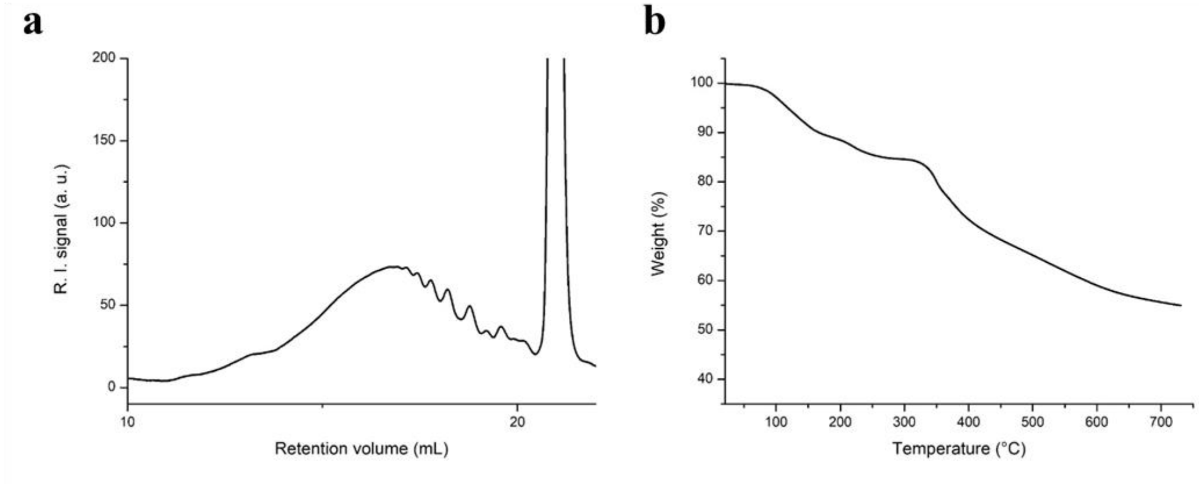

Figure S17: a) SEC trace of **P6** in THF, R. I. detection. **b)** TGA curve of **P6** at 10 °C/min under N_2.

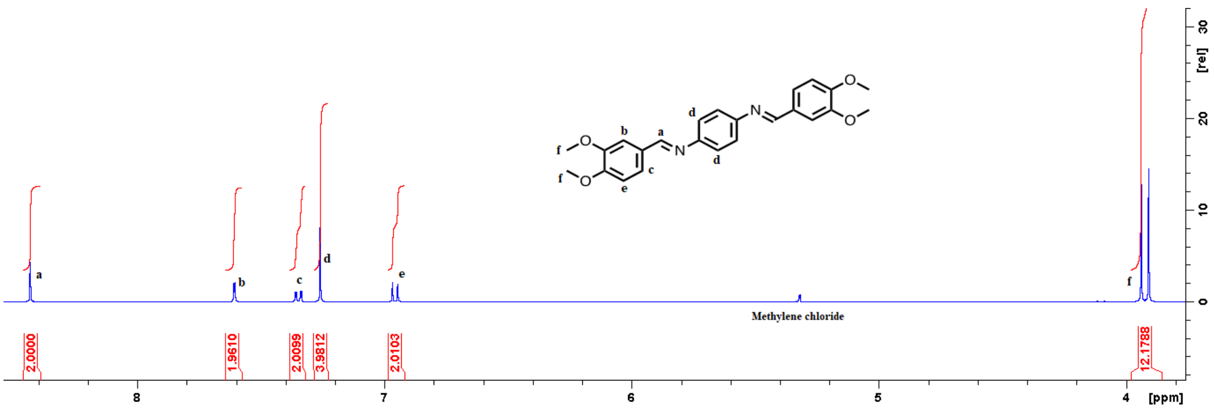

Figure S18: ^{1}H NMR spectrum of **M1a** (400.20 MHz, in CD_2Cl_2).

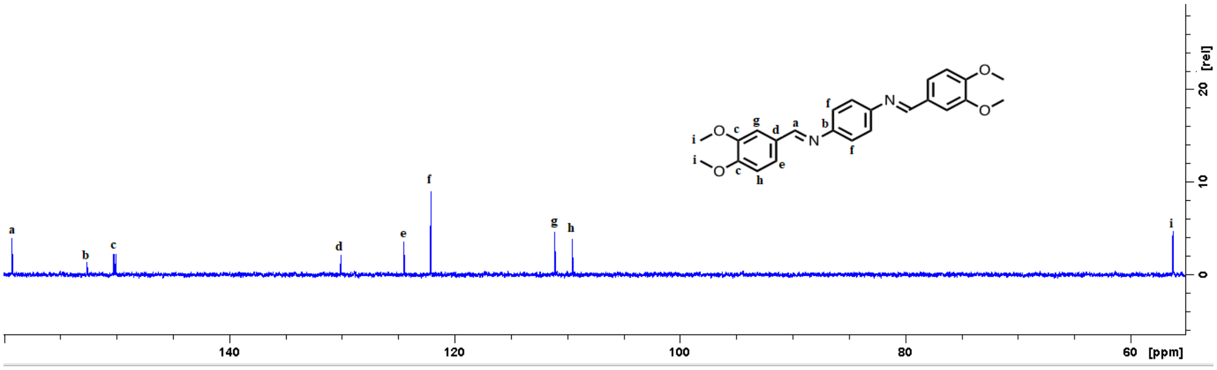

Figure S19: ^{13}C NMR spectrum of **M1a** (100.70 MHz, in CD_2Cl_2).

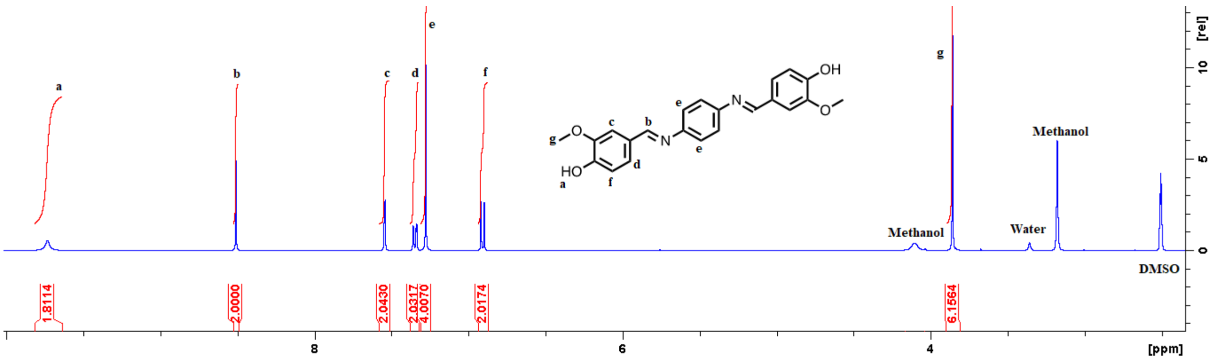

Figure S20: ^{1}H NMR spectrum of **M1b** (400.20 MHz, in DMSO-d_6).

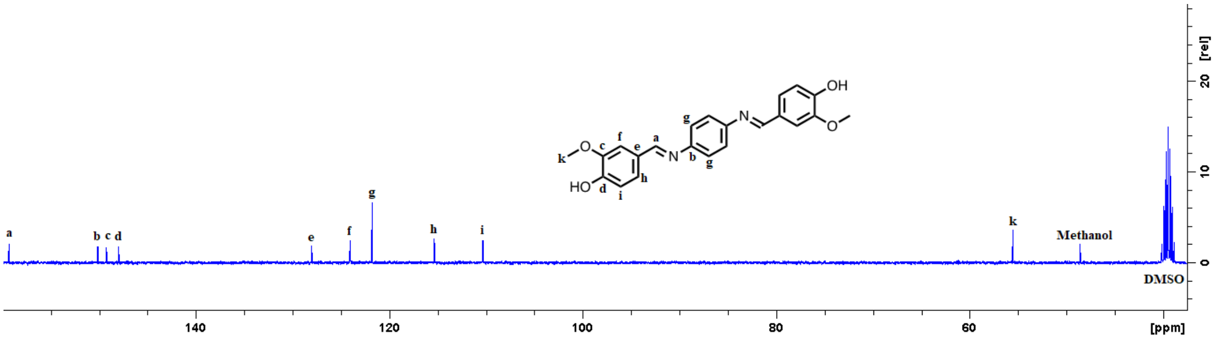

Figure S21: ^{13}C NMR spectrum of **M1b** (100.70 MHz, in DMSO-d_6).

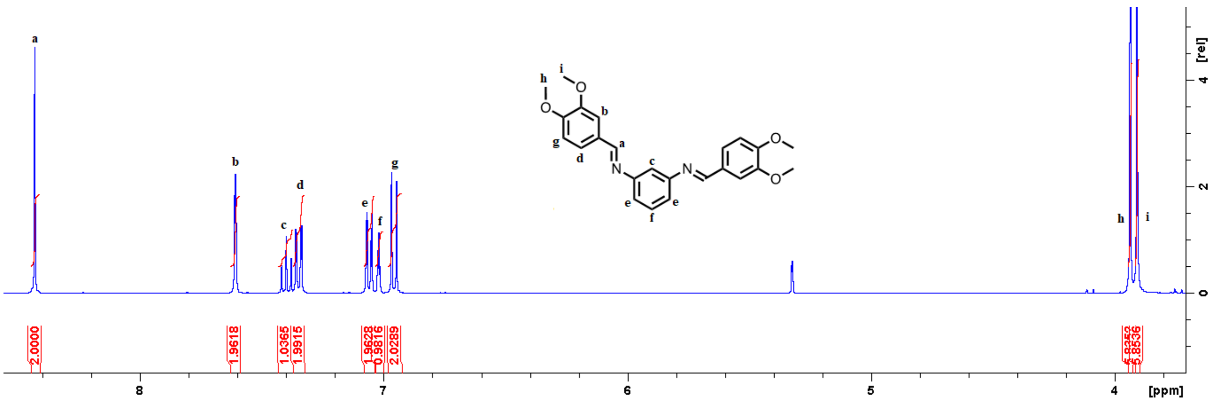

Figure S22: ^{1}H NMR spectrum of **M2** (400.20 MHz, in CD_2Cl_2).

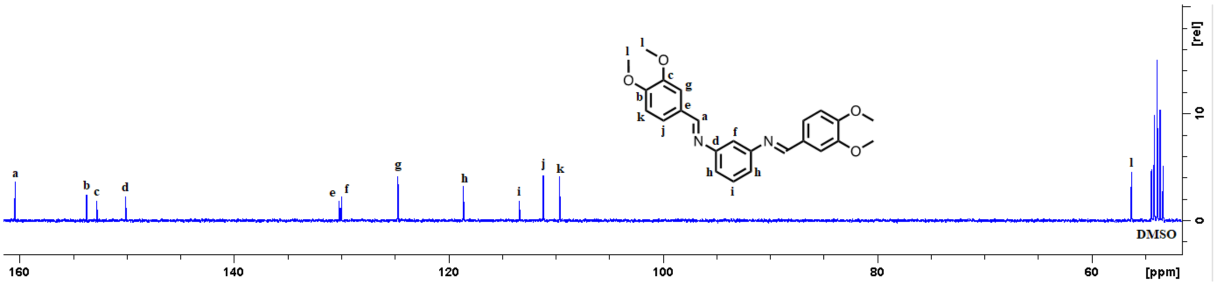

Figure S23: ^{13}C NMR spectrum of **M2** (100.70 MHz, in CD_2Cl_2).

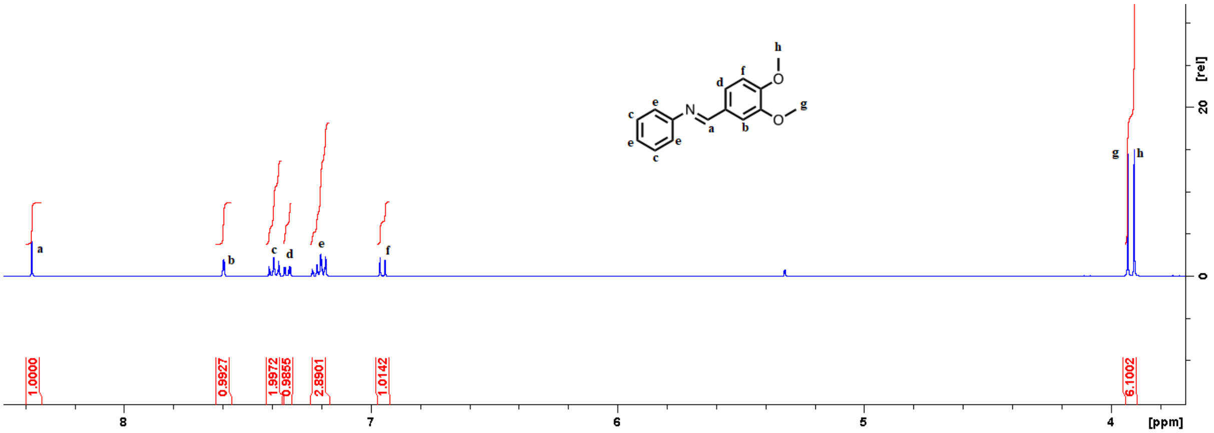

Figure S24: ^{1}H NMR spectrum of **M3a** (400.20 MHz, in CD_2Cl_2).

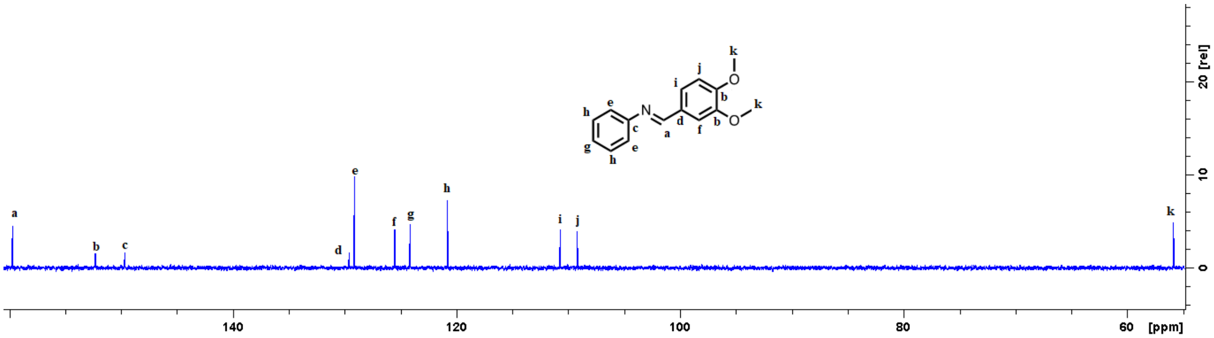

Figure S25: ^{13}C NMR spectrum of **M3a** (100.70 MHz, in CD_2Cl_2).

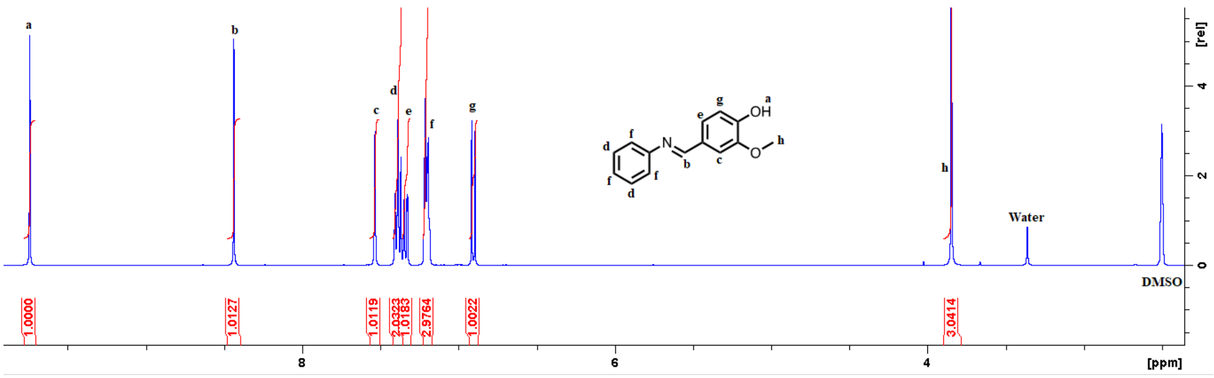

Figure S26: ^{1}H NMR spectrum of **M3b** (400.20 MHz, in DMSO-d_6).

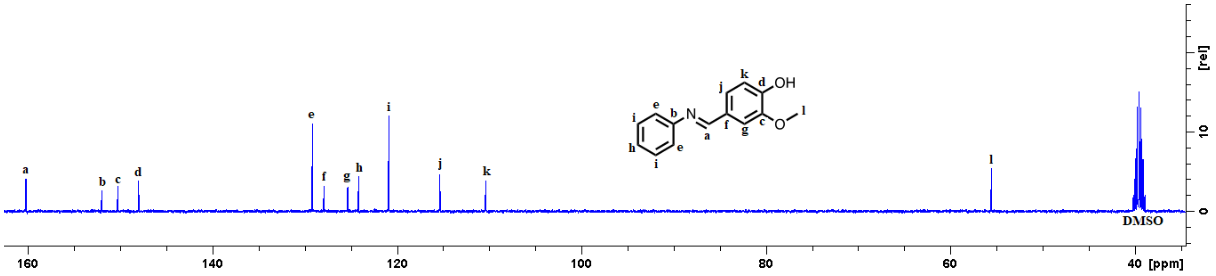

Figure S27: ^{13}C NMR spectrum of **M3b** (100.70 MHz, in DMSO-d_6).

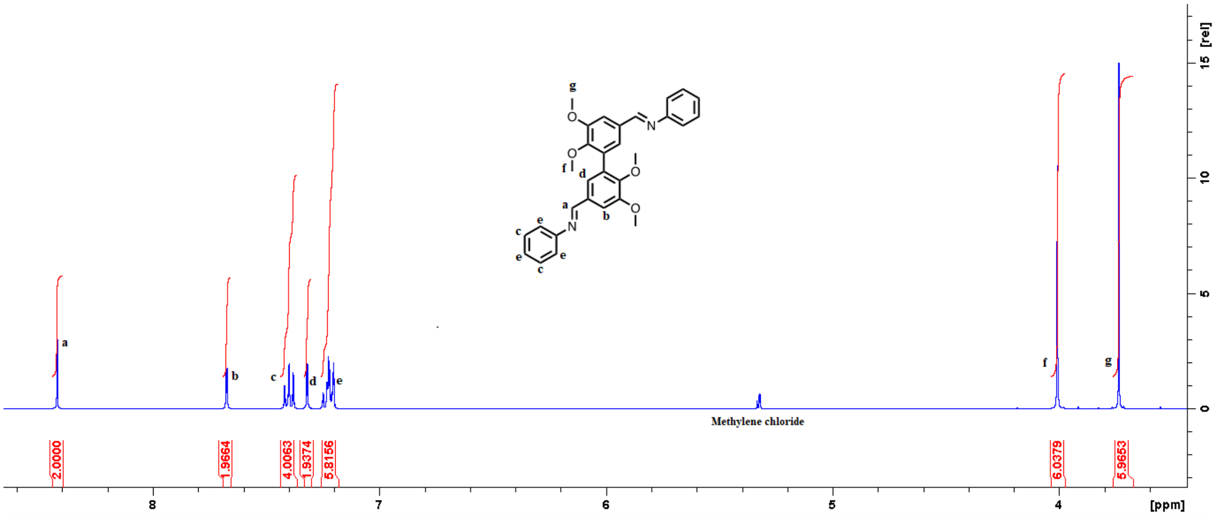

Figure S28: ^{1}H NMR spectrum of **M4** (400.20 MHz, in CD_2Cl_2).

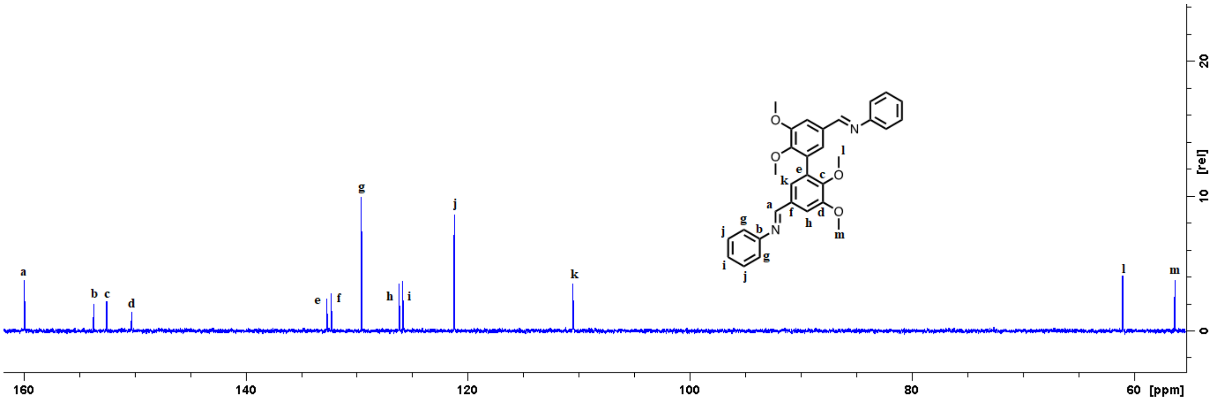

Figure S29: ^{13}C NMR spectrum of **M4** (100.70 MHz, in CD_2Cl_2).

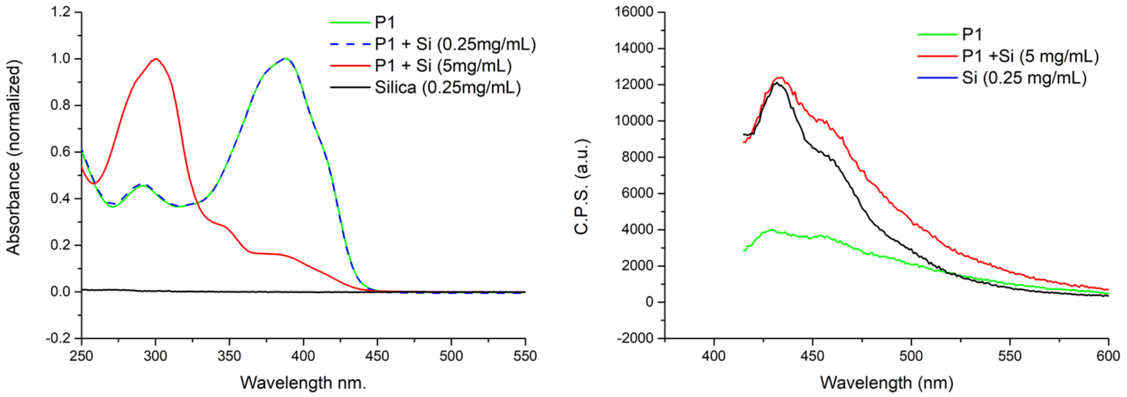

Figure S30: Absorbance spectra of **P1** in methylene chloride (10^{-2} g/L) with different amount of silica and emission spectra of **P1** (10^{-3} g/L, excitation at 360 nm) with and without silica. All solutions were filtered before analysis.

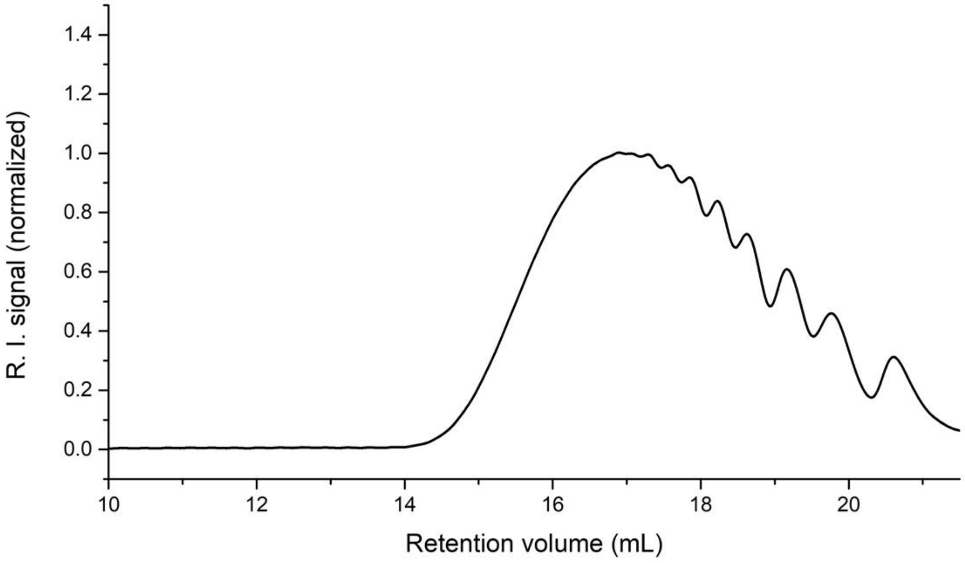

Figure S31: SEC trace of a **P4** polymer synthesized by only performing a "recovery step" on a stoichiometric mixture of monomers (R.I. detection, in THF).

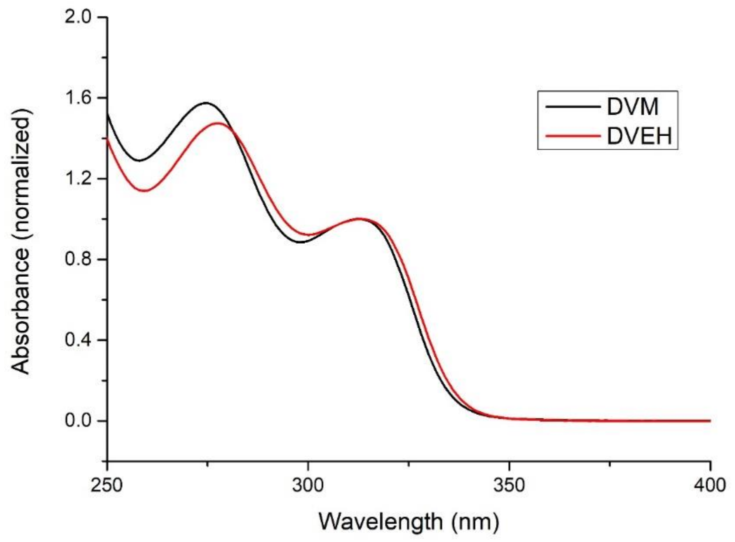

Figure S32: Absorbance spectra of **DVEH** and **DVM** in methylene chloride, 10^{-2} g/L.

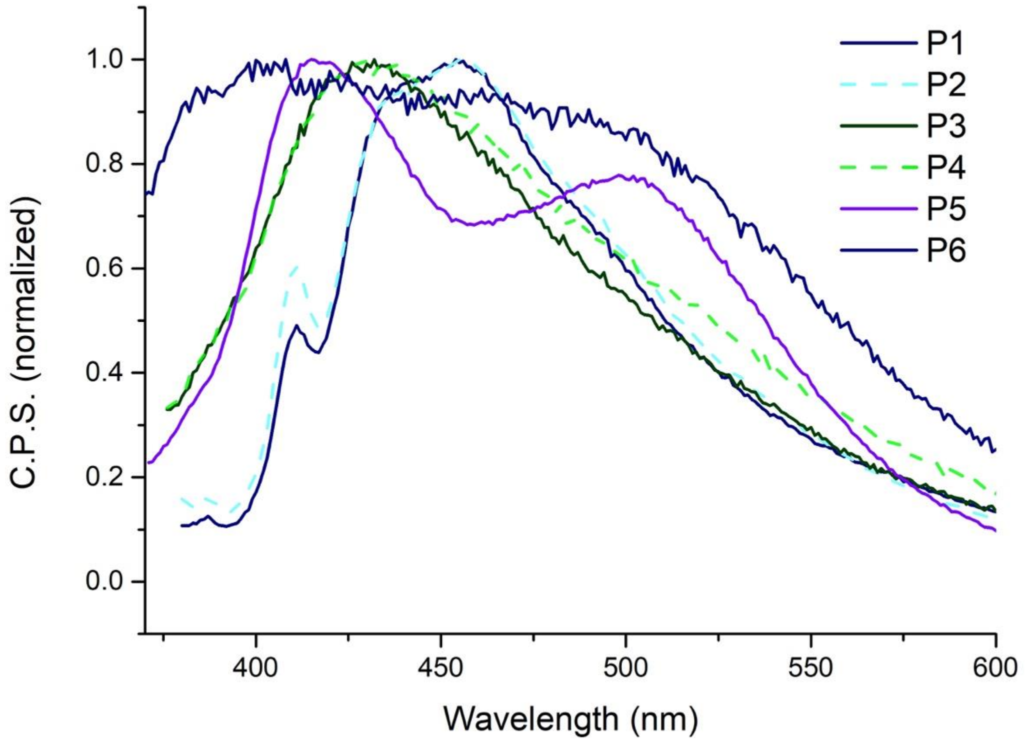

Figure S33: Emission spectra of polyazomethines in methylene chloride and their structure (10^{-2} g/L, which corresponds to an absorbance of 1, soluble fraction for **P5**, integration time: 0.5 s).

Table S1: Absorbance characterizations of model compounds and corresponding polymers

Molecule model or Polyazomethine	Absorbance (nm)[a]	ε (cm^{-1}. mol^{-1}. L^{-1})[b]
**M1a**	363 - 285	34 400
**M1b**	ND	ND
**M2**	328 - 284	29 600
**M3a**	323 - 283	22 700
**M3b**	ND	ND
**M4**	323 - 281	44 000
**P4**	363 - 286	293 000
**P6**	326 - 284	139 000

[a] Determined in methylene chloride, 10^{-2} g/L. [b] Measured in methylene chloride by using a range of concentrations from 10^{-2} to 10^{-3} g/L, at the maximum of absorbance.

Table S2: Crystal data and structure refinement for M3b. **Deposition Number 2113008**

Empirical formula	$C_{28}H_{26}N_2O_4$
Formula weight	454.51
Temperature/K	120
Crystal system	orthorhombic
Space group	C2221
a/Å	16.7830(3)
b/Å	18.1688(3)
c/Å	15.2042(3)
α/°	90
β/°	90
γ/°	90
Volume/Å^3	4636.17(15)
Z	8
ρcalcg/cm^3	1.302
μ/mm^{-1}	0.707
F(000)	1920
Radiation	Cu Kα (λ = 1.54184)
2Θ range for data collection/°	7.17 to 147.204
Reflections collected	33211
Independent reflections	4643 [R_{int} = 0.1079, R_{sigma} = 0.0419]
Data/restraints/parameters	4643/0/313
Goodness-of-fit on F2	1.098
Final R indexes [I>=2σ (I)]	R1 = 0.0677, wR2 = 0.1684
Final R indexes [all data]	R1 = 0.0682, wR2 = 0.1687
Flack parameter	-0.1(4)

Table S3: Crystal data and structure refinement for M1a. **Deposition Number 2113009**

Empirical formula	$C_{24}H_{24}N_2O_4$
Formula weight	404.45
Temperature/K	120
Crystal system	triclinic
Space group	P-1
a/Å	6.7944(3)
b/Å	6.9613(4)
c/Å	11.7543(7)
α/°	77.347(5)
β/°	81.596(4)
γ/°	65.069(5)
Volume/Å^3	490.99(5)
Z	1
ρcalcg/cm^3	1.368
μ/mm^{-1}	0.761\\\\
F(000)	214
Radiation	Cu Kα (λ = 1.54184)
2Θ range for data collection/°	7.722 to 147.994
Reflections collected	6943
Independent reflections	1948 [R_{int} = 0.0161, R_{sigma} = 0.0109]
Data/restraints/parameters	1948/0/139
Goodness-of-fit on F2	1.071
Final R indexes [I>=2σ (I)]	R1 = 0.0360, wR2 = 0.1046
Final R indexes [all data]	R1 = 0.0367, wR2 = 0.1056

Table S4: Crystal data and structure refinement for M3a. **Deposition Number 2113010**

Empirical formula	$C_{15}H_{15}NO_2$
Formula weight	241.28
Temperature/K	120
Crystal system	orthorhombic
Space group	Pbca
a/Å	15.8747(3)
b/Å	6.14640(10)
c/Å	25.5742(5)
α/°	90
β/°	90
γ/°	90
Volume/Å3	2495.33(8)
Z	8
ρcalcg/cm^3	1.284
μ/mm^{-1}	0.686
F(000)	1024.0
Radiation	Cu Kα (λ = 1.54184)
2Θ range for data collection/°	6.912 to 147.904
Reflections collected	16515
Independent reflections	2513 [R_{int} = 0.0351, R_{sigma} = 0.0125]
Data/restraints/parameters	2513/0/166
Goodness-of-fit on F2	1.061
Final R indexes [I>=2σ (I)]	R1 = 0.0390, wR2 = 0.1012
Final R indexes [all data]	R1 = 0.0394, wR2 = 0.1029

Table S5: Crystal data and structure refinement for M4. **Deposition Number 2113011**

Empirical formula	$C_{30}H_{28}N_2O_4$
Formula weight	480.54
Temperature/K	120
Crystal system	monoclinic
Space group	P21/c
a/Å	7.44380(10)
b/Å	17.1323(2)
c/Å	19.4003(2)
α/°	90
β/°	93.7320(10)
γ/°	90
Volume/Å^3	2468.86(5)
Z	4
ρcalcg/cm^3	1.293
μ/mm^{-1}	0.693
F(000)	1016.0
Radiation	Cu Kα (λ = 1.54184)
2Θ range for data collection/°	6.89 to 148.284
Reflections collected	19039
Independent reflections	4934 [R_{int} = 0.0271, R_{sigma} = 0.0219]
Data/restraints/parameters	4934/0/330
Goodness-of-fit on F2	1.040
Final R indexes [I>=2σ (I)]	R1 = 0.0389, wR2 = 0.1012
Final R indexes [all data]	R1 = 0.0458, wR2 = 0.1071

Table S6 Crystal data and structure refinement for M1b. **Deposition Number 2113012**

Empirical formula	$C_{24}H_{28}N_2O_6$
Formula weight	440.48
Temperature/K	120
Crystal system	monoclinic
Space group	P21/n
a/Å	8.4611(2)
b/Å	7.6710(2)
c/Å	17.4975(3)
α/°	90
β/°	101.566(2)
γ/°	90
Volume/Å^3	1112.62(4)
Z	2
ρcalcg/cm^3	1.315
μ/mm^{-1}	0.751
F(000)	468.0
Radiation	Cu Kα (λ = 1.54184)
2Θ range for data collection/°	10.32 to 150.098
Reflections collected	7388
Independent reflections	2256 [R_{int} = 0.0252, R_{sigma} = 0.0163]
Data/restraints/parameters	2256/0/150
Goodness-of-fit on F2	1.117
Final R indexes [I>=2σ (I)]	R1 = 0.0537, wR2 = 0.1355
Final R indexes [all data]	R1 = 0.0539, wR2 = 0.1357

Table S7 Crystal data and structure refinement for M2. **Deposition Number 2113013**

Empirical formula	$C_{24}H_{24}N_2O_4$
Formula weight	404.45
Temperature/K	120
Crystal system	monoclinic
Space group	P21/c
a/Å	26.6983(2)
b/Å	8.67810(10)
c/Å	8.93320(10)
α/°	90
β/°	91.4800(10)
γ/°	90
Volume/Å^3	2069.05(4)
Z	4
ρcalcg/cm^3	1.298
μ/mm^{-1}	0.722
F(000)	856.0
Radiation	Cu Kα (λ = 1.54184)
2Θ range for data collection/°	6.624 to 149.762
Reflections collected	15815
Independent reflections	4173 [R_{int} = 0.0165, R_{sigma} = 0.0124]
Data/restraints/parameters	4173/0/276
Goodness-of-fit on F2	1.057
Final R indexes [I>=2σ (I)]	R1 = 0.0342, wR2 = 0.0948
Final R indexes [all data]	R1 = 0.0352, wR2 = 0.0957
